# Assessment of insulin resistance by a ^13^C glucose breath test: a new tool for early diagnosis and follow-up of high-risk patients

**DOI:** 10.1186/1475-2891-9-25

**Published:** 2010-05-27

**Authors:** Meir Mizrahi, Gadi Lalazar, Tomer Adar, Itamar Raz, Yaron Ilan

**Affiliations:** 1 Liver Unit, Department of Medicine, Hebrew University-Hadassah Medical Center (Ein Karem), Jerusalem (12000), Israel; 2 Diabetes Unit, Department of Medicine, Hebrew University-Hadassah Medical Center (Ein Karem), Jerusalem (12000), Israel

## Abstract

**Background/Aims:**

Insulin resistance (IR) plays an important role in the pathogenesis of diabetes and non-alcoholic fatty liver disease (NAFLD). Current methods for insulin resistance detection are cumbersome, or not sensitive enough for early detection and follow-up. The BreathID^® ^system can continuously analyse breath samples in real-time at the point-of-care. Here we determined the efficacy of the BreathID^® ^using the ^13^C-Glucose breath test (GBT) for evaluation of insulin resistance.

**Methods:**

Twenty healthy volunteers were orally administered 75 mg of ^13^C-glucose 1-^13^C. An oral glucose tolerance test (OGTT) was performed immediately; followed by serum glucose and insulin level determinations using GBT. GBT and OGTT were repeated following exercise, which alters insulin resistance levels.

**Results:**

Within-subject correlations of GBT parameters with serum glucose and serum insulin levels were high. Before and after exercise, between-subjects correlations were high between the relative insulin levels and the % dose recoveries at 90 min (PDR 90), and the cumulative PDRs at 60 min (CPDR 60). Pairwise correlations were identified between pre-exercise Homeostasis Model Assessment (HOMA) IR at 90 min and PDR 90; HOMA B (for beta cell function) 120 and CPDR 30; HOMA IR 60 and peak time post-exercise; and HOMA B 150 with PDR 150.

**Conclusions:**

The non-invasive real-time BreathID^® ^GBT reliably assesses changes in liver glucose metabolism, and the degree of insulin resistance. It may serve as a non-invasive tool for early diagnosis and follow up of patients in high-risk groups.

## Introduction

Insulin resistance (IR) is defined as a subnormal response to both endogenous and exogenous insulin [1]. It is characterized by decreasing sensitivity of target tissues to the action of insulin, by elevated blood glucose concentration, and increased hepatic production of atherogenic lipids[2,3]. Insulin resistance contributes to the pathophysiology of diabetes, and is a hallmark of obesity, metabolic syndrome, and many cardiovascular diseases [4-14]. Obesity, mainly of the abdominal type, is associated with resistance to the effects of insulin on peripheral glucose and fatty acid utilization, often leading to type 2 diabetes mellitus [3,15-17].

Quantifying insulin sensitivity/resistance in humans and animal models is important for basic and clinical science, and eventually for clinical practice [6]. Direct and indirect methods of varying complexity are currently employed. Some rely on steady-state analysis of glucose and insulin, while others rely on dynamic testing [6]. Each of these methods has distinct advantages and limitations.

There is no consensus on when or how to evaluate patients at risk for altered insulin resistance. In a clinical setting, it would be useful to identify obese patients who are insulin resistant prior to development of overt diabetes. This group is at the highest risk for developing type 2 diabetes mellitus, cardiovascular disease, and non-alcoholic fatty liver disease.

Breath testing is based on the principal that an ingested substrate is metabolized, and a measurable metabolite is then expelled by the respiratory system. Breath testing has been used experimentally and clinically for several decades [12], including for follow-up on patients with chronic liver disorders. The major drawbacks of these tests are: the need for traditional, cumbersome isotopic ratio mass spectrometry methods, a prolonged testing time, high cost, and patient inconvenience. The BreathID^® ^continuous online ^13^C-methacetin breath test (MBT), which reflects hepatic microsomal function (CYP1A2), is a laser-based technology that creates an infrared emission precisely matching the absorption spectrum of CO_2_. MBT can detect variations of less than 1/1000 in the ^13^CO_2_/^12^CO_2 _ratio measurement,^18 ^measuring CO_2 _by molecular correlation spectroscopy. This test offers several advantages. It is an office-based, non-invasive tool for the assessment of substrate metabolism. It does not involve a blood test, and it can provide immediate results at the point-of-care [18].

The aim of the present study was to determine the efficacy of the BreathID^® ^using the ^13^C-Glucose breath test (GBT) as a tool for evaluation of insulin resistance.

## Methods

### Healthy Volunteers

Twenty healthy volunteers were enrolled in the study. There were 11 males and 9 females with a mean age of 24.85 yr (19-33 yr), mean body mass index (BMI) of 27.04 (19.89-38.74), mean waist circumference of 92.5 cm (61-121 cm), mean weight of 80.07 kg (49-121), and mean height of 171.31 cm (159-188 cm). All participants were screened by medical history and physical examination. None had a history of active or previous diabetes, liver disease, or alcohol or drug abuse, or were taking medications. All participants gave written informed consent to their participation in the study, which was conducted in strict adherence to the principles of the Declaration of Helsinki. The institutional Ethics Committee approved all experiments.

### Oral glucose tolerance test combined with non-invasive breath testing

Following an over-night (at least 8-hr) fast, all subjects were connected to the breath-testing unit's BreathID^® ^system (Exalenz Bioscience Ltd., Modiin, Israel) via nasal cannula (IDcircuit™) and were orally administered 75 mg of ^13^C-glucose 1-^13^C (Isotec) dissolved in 150 mL of water with 75 g glucose. Breath samples were collected simultaneously with follow up of serum glucose and insulin levels (at 0, 30, 60, 90, 120, 150, and 180 min) using an automatic breath sampling unit under continuous capnographic control, for 180 minutes after the labelled substrate was administered to the patient. The ^13^CO_2_/^12^CO_2 _ratios in the breath samples were frequently determined and mapped on the screen (once every 2-3 min the machine measured the ^13^CO_2_/^12^CO_2 _calculating the PDR at the same point). During the test period, all volunteers continued fasting and were at rest, to eliminate any variability in CO_2 _excretion due to the ingestion of food or physical activity.

### Analysis of breath-test data

Results obtained from the device were expressed as percentage dose recovered (PDR-expresses the rate of substrate metabolism) of administered ^13^C [%/h], cumulative percentage dose of ^13^C recovered (CPDR-area under the curve) [%] over time (at 0, 30, 60, 90, 120, 150, and 180 min after ingestion of labelled glucose), PDR peak, and the time to peak. The change in the 13C/12C ratio takes the specific test details into account, normalizing the results, and making them independent of differences in weight, height, dose, or substrate type and purity[14,15]. CPDR is the numeric integral of PDR and describes the total amount of substrate metabolized at any given time. Data are expressed in units of %/hr for PDR, and % for CPDR. The BreathID^® ^device plots the PDR and CPDR in real time, and provides PDR peak value and peak time.

### Determining the sensitivity of the test for detection of insulin resistance

We used exercise to assess the sensitivity of the GBT to follow-up changes in insulin resistance. Both oral GTT and the GBT were repeated before and after physical exercise (30 min of walking at 6 km/hr). Subjects were followed for ^13^CO_2_/^12^CO_2 _ratios, and serum glucose and insulin levels (at 0, 30, 60, 90, 120, 150, and 180 min).

### Statistical analysis

The comparison of two independent groups used Student's *t *test, and the non-parametric Mann-Whitney test. The association between two variables was assessed by calculating the Pearson and the Spearman correlation coefficients. Calculations of the within-subjects correlation coefficients between each pair over all subjects were derived from an analysis of variance table in a General Linear Model, or from the within-cell correlations in Multivariate Analysis of Variance (ANOVA). All tests were two-tailed, and a *p *value of 0.05 or less was considered statistically significant.

### HOMA score

The Homeostasis Model Assessment (HOMA) is designed to predict the homeostatic concentrations of fasting insulin and glucose, which arise from varying degrees of beta-cell deficiency and insulin resistance. The model is nonlinear, but can be simply approximated. Two types of HOMA scores are currently being evaluated in clinical practice for determining fasting glucose and insulin levels. HOMA IR = insulin resistance = (fasting insulin in mU/L) × (fasting plasma glucose in mmol/L)/22.5. HOMA B = beta-cell function [%] = 20 × (fasting insulin in mU/L)/((fasting glucose in mmol/L) - 3.5). We calculated the two types of HOMA scores following glucose ingestion at 0, 30, 60, 90, 120, 150, and 180 min.

### QUICKI score

The quantitative insulin sensitivity check index (QUICKI) correlates well with the hyperinsulinemic euglycemic clamp technique. The inverse of QUICKI is the insulin resistance index, which is a good indirect measure of insulin resistance. We calculated the QUICKI score for serum glucose and insulin levels 0, 30, 60, 90, 120, 150, and 180 min following glucose ingestion, using the following formulas: QUICKI = 1/(log_10 _((fasting insulin in ÂμU/mL) × (fasting plasma glucose in mg/dL))) = 1/(log_10 _(fasting insulin in ÂμU/mL) + log_10 _(fasting plasma glucose in mg/dL)). Insulin resistance index (IR) = log_10 _(fasting insulin) + log_10 _(fasting plasma glucose) = 1/(QUICKI).

## Results

### Glucose breath test correlates with serum glucose levels

**Figure 1:**
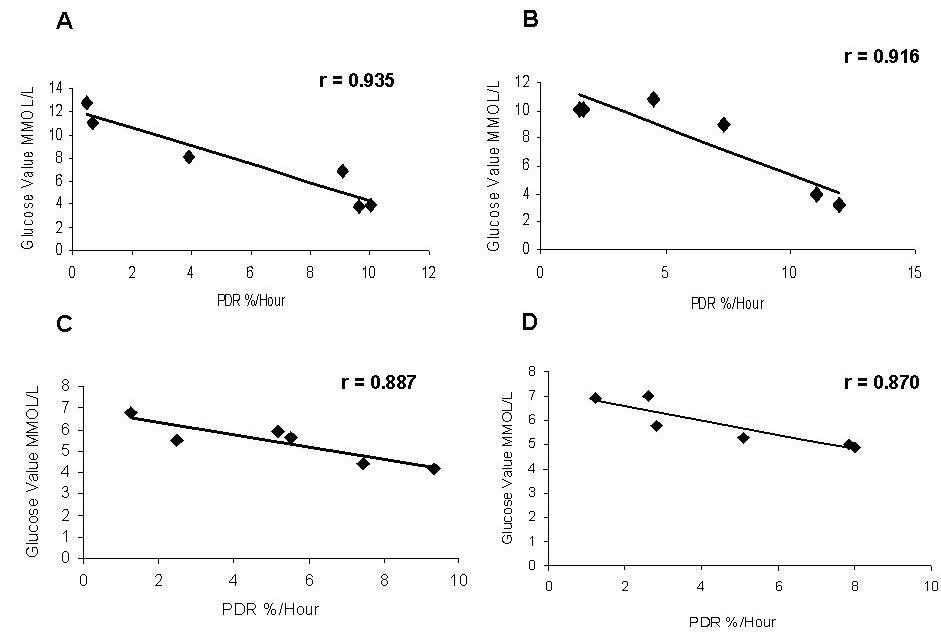
**Patients underwent oral glucose tolerance test and glucose breath test**. Correlation between serum glucose levels in the oral glucose tolerance test and PDR in a patient with BMI > 30 (A) before exercise; (B) after exercise; and for a patient with BMI < 30 (C) before exercise; and (D) after exercise.

We found a high correlation between GBT parameters and serum glucose levels. Fig. 1A shows the negative correlation between glucose levels in the OGGT test and the PDR (r = 0.935) before exercise, in a patient with a BMI > 30. Physical exercise is known to improve the degree of insulin resistance, and was used in the present study to assess the sensitivity of the GBT for changes in the degree of insulin resistance. Alterations in the degree of IR post exercise were detected by the GBT. Fig. 1B shows the negative correlation between glucose levels in the OGGT test and the PDR (r = 0.916) after exercise in this patient.

Similar correlations were found for patients with BMIs < 30. Fig. 1C shows a high negative correlation between glucose levels in the OGGT test and the PDR (r = 0.887) before exercise in a patient with a BMI < 30. Fig. 1D shows the negative correlation between glucose levels in the OGGT test and the PDR (r = 0.870) after exercise in a patient with BMI < 30.

**Figure 2:**
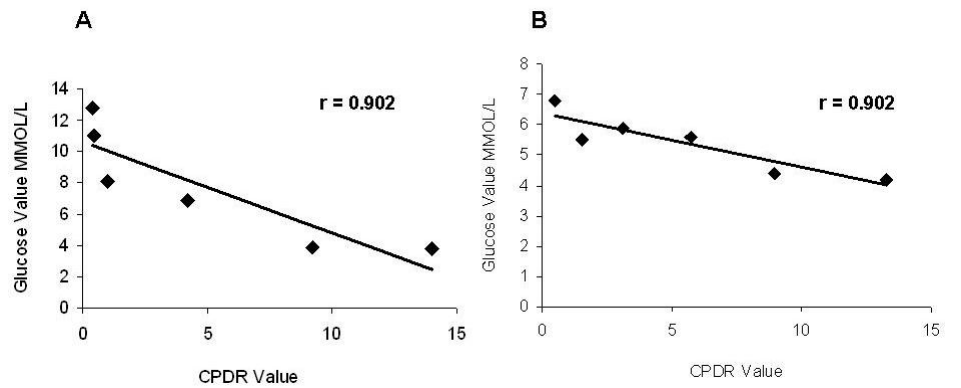
**Correlations were calculated between glucose levels and CPDR in (A) a patient with BMI > 30; and (B) in a patient with BMI < 30**. Each dot represents a point of time following oral administration of glucose.

Like the PDR, the CPDR, the cumulative percentage dose of ^13^C recovered over time, negatively correlated with glucose levels. Fig. 2A shows the negative correlation between glucose levels and the CPDR ratio (r = 0.813) in a patient with a BMI > 30. Fig. 2B shows the negative correlation between glucose levels and the CPDR ratio (r = 0.813) in a patient with a BMI > 30.

### Glucose breath test correlates with serum insulin levels

**Figure 3:**
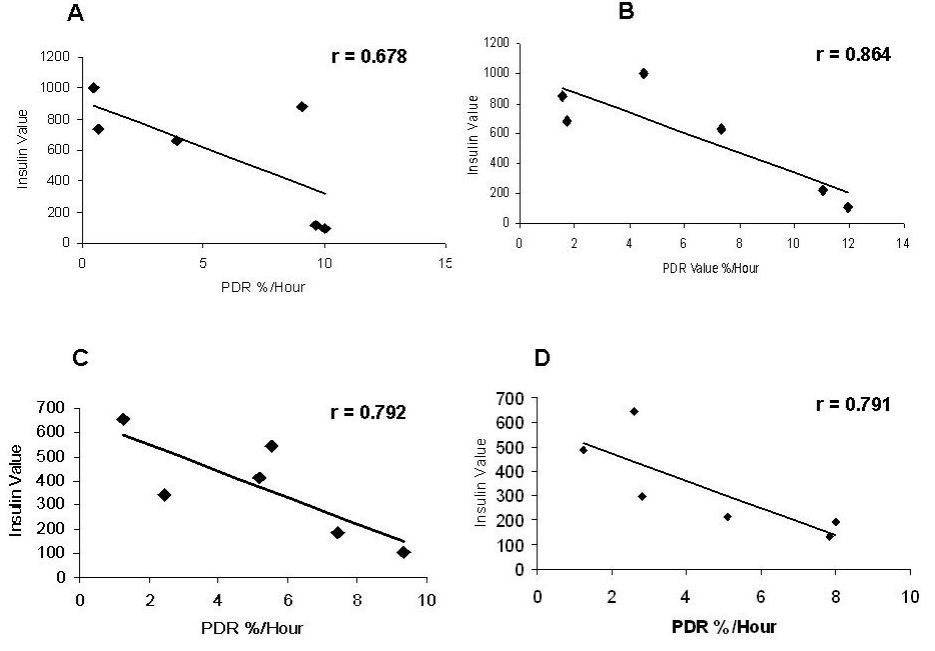
**Correlations between serum insulin levels and PDR in (A) a patient with BMI > 30 before exercise; and (B) after exercise; and (C) in a patient with BMI < 30 before exercise; and (D) after exercise**.

We found a high negative correlation between GBT parameters and serum insulin levels. Fig. 3A shows the negative correlation in the OGGT test before exercise between insulin levels and the PDR (r = 0.678) in a patient with a BMI > 30. A high negative correlation with insulin levels was also noted after exercise. Fig. 3B shows the negative correlation between insulin levels and the PDR rate (r = 0.864) in the OGGT test after exercise in this patient.

Similar correlations were found in patients with BMI < 30. Fig. 3C shows the negative correlation between serum insulin levels and the PDR (r = 0.792) in the OGGT test before exercise in a patient with a BMI < 30. A similar high negative correlation remained after exercise. Fig. 3D shows the negative correlation between glucose level and the PDR rate (r = 0.791) in the OGGT test after exercise in this patient.

**Figure 4:**
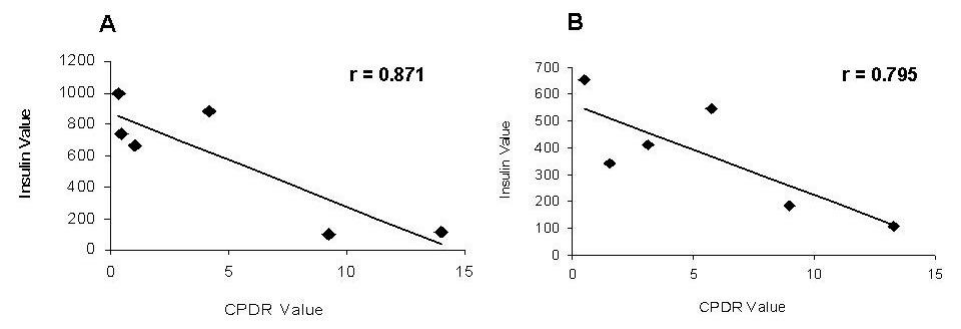
**Correlations between insulin levels and CPDR in (A) a patient with BMI > 30; and in (B) a patient with BMI < 30**. Each dot represents a point of time following oral administration of glucose.

Like the PDR, the CPDR was negatively correlated with serum insulin levels. Fig. 4A shows the negative correlation between insulin levels and the CPDR ratio in a patient with BMI > 30 (r = 0.871), while Fig. 4B, shows the correlation in a patient with BMI < 30 (r = 0.795).

### Determining the sensitivity of the GBT for assessment of insulin resistance

**Figure 5:**
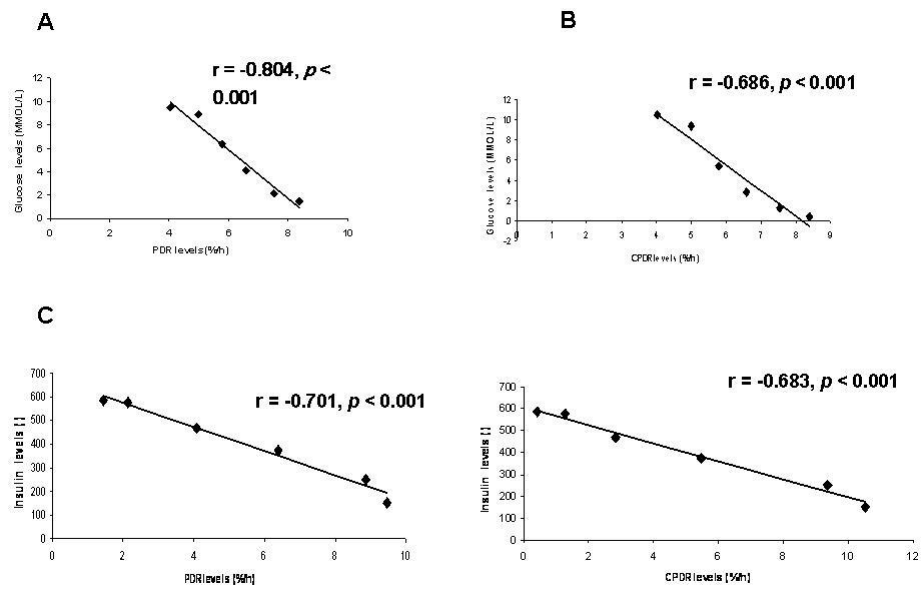
**Correlations between the median value of glucose levels for all patients together every 30 minutes during OGTT and PDR every 30 min**. in (A) and (B) for CPDR every 30 min; and correlations between the median value of insulin levels for all patients together every 30 minutes during OGTT and PDR every 30 min. (C); and (D) for CPDR every 30 min.

A high within-subject correlation between serum glucose levels and GBT is shown in Table 1. High negative correlations were noted for the PDR (r = -0.804, *p *< 0.001), and for the CPDR (r = -0.686, *p *< 0.001), Fig. 5A and 5B respectively. Similarly, high within-subject negative correlations between serum insulin levels and GBT were found. High negative correlations were detected for PDR (r = -0.701, *p *< 0.001) and for CPDR (r = -0.683, *p *< 0.001) fig. 5C and 5D respectively.

**Table 1:** Within-subject correlations between serum glucose and insulin levels, and GBT percentage dose recovery (PDR), and cumulative percentage dose recovery (CPDR) before and after exercise.

	PDR - Glucose		PDR - Insulin		CPDR - Glucose		CPDR - Insulin	
	R	*p*	R	*p*	R	*p*	R	*p*
**Before exercise**	-0.804	< 0.001	-0.701	< 0.001	-0.686	< 0.001	-0.683	< 0.001
**After exercise**	-0.757	< 0.001	-0/709	< 0.001	-0.715	< 0.001	-0.636	< 0.001

To further determine the sensitivity of the test, GBT and OGTT were repeated following physical exercise, which alters the level of insulin resistance. High within-subject negative correlations of the GBT parameters with serum glucose levels were found (PDR r = -0.757, *p *< 0.001; CPDR r = -0.715, *p *< 0.001) and with serum insulin levels (PDR r = -0.709, *p *< 0.001; CPDR r = -0.636, *p *< 0.001).

High between-subjects correlations were found between the insulin levels ratio and the PDR 90 ratio (r = 0.8, *p *< 0.001) before and after exercise, and between the ratio of glucose levels and the CPDR 60 ratios (r = 0.92, *p *< 0.001) before and after exercise.

This data suggests that the GBT may serve as a valid tool for detection of mild alterations in insulin resistance.

### Glucose breath test correlates with HOMA score

**Figure 6:**
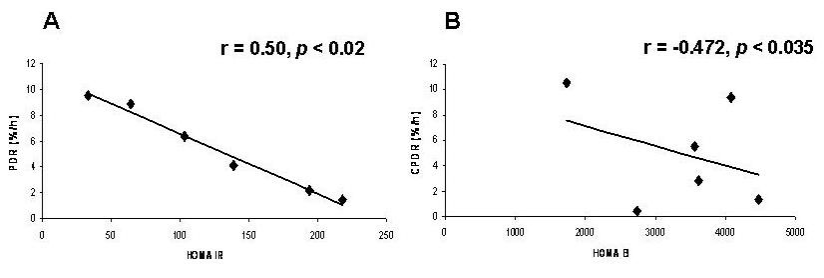
**Correlations between the median value of HOMA IR for all patients together every 30 minutes during OGTT and PDR every 30 min**. in (A) and (B) for CPDR and HOMA B every 30 min.

High negative correlations were found before exercise between the HOMA IR and the PDR value (Fig 6A). At 90 min the correlation was the highest for HOMA IR(r = -0.479, *p *< 0.032), and at 120 min, for HOMA B and CPDR, 30 min (r = -0.472, *p *< 0.035). Similarly, negative correlations between the HOMA IR at 60 min and peak time post exercise (r = 0.50, *p *< 0.02), and between the HOMA B at 150 min with the PDR at 150 min (r = -0.653, *p *< 0.002) were identified.

### Glucose breath test correlates with QUICKI score

High correlations were detected before exercise between the QUICKI score and the PDR at 120 min (*p *< 0.032), between the QUICKI score and the peak time (*p *< 0.05), and post exercise between the PDR at 90 min and the QUICKI (*p *< 0.05).

### Correlations between the GBT and age, gender, and BMI

GBT data was analyzed as a function of age, gender, and BMI, using multiple linear regressions. For the non-obese (BMI < 30) subgroup (*n *= 14) post exercise peak time (the time to get to the higher point of ^13^CO_2_/^12^CO_2 _ratio) was the most significant variable (not shown).

## Discussion

Our data suggest that the non-invasive real-time BreathID^® ^GBT reliably assesses changes in the liver glucose metabolism, and may serve as a tool for determining insulin resistance. High within-subject correlations between serum glucose and GBT were detected. The GBT parameters highly correlated with serum insulin levels.

In order to assess the sensitivity of the GBT for detecting insulin resistance, the study was performed in healthy volunteers after exercise. Exercise is a key component for the successful management of many obesity-related metabolic complications, including insulin resistance.^19 ^Both chronic and acute endurance exercise has an effect on insulin action in obesity. Exercise-induced alterations in fatty acid partitioning within muscle cells affect insulin sensitivity [19]. GBT and OGTT were repeated following physical exercise. High between-subjects correlations were found between the insulin levels ratio and the PDR 90 ratio before and after exercise, and between the ratio of glucose levels and the CPDR 60 ratio before and after exercise. Correlations were identified before exercise between the HOMA IR at 90 min and the PDR at 90 min, and between the HOMA B at 120 min and the CPDR at 30 min. Correlations were identified after exercise between the HOMA IR at 60 min and the peak time, and between the HOMA B at 150 min and the PDR at 150 min. This data suggests that the GBT may serve as a valid tool for detection of mild alterations in insulin resistance.

Currently available methods for assessment of insulin resistance are invasive and cumbersome, making them impractical for use at the point-of-care. The gold standard diagnostic test for insulin resistance is the hyperinsulinemic-euglycemic clamp, but this method is unsuitable for everyday clinical use. The hyperinsulinemic euglycemic clamp and the insulin suppression test, which are both labour and time intensive, directly assess insulin-mediated glucose utilization under steady-state conditions [20,21]. The degree of insulin resistance is inversely proportional to the glucose uptake by target tissues during the procedure. A slightly less complex indirect method relies on minimal model analysis of a frequently sampled intravenous glucose tolerance test. The insulin sensitivity test (IST) and the Insulin tolerance test (ITT) measure the decline in serum glucose after an IV bolus of regular insulin. They primarily assess the insulin-stimulated uptake of glucose into skeletal muscle [21-25]. ISI is calculated for fat-free body mass by dividing the glucose disposal rate by the average plasma insulin concentration [6].

Surrogate indices for insulin sensitivity/resistance, including QUICKI, HOMA, 1/insulin, and Matusda index, are all derived from blood insulin and glucose concentrations under fasting conditions (steady state) or after an oral glucose load (dynamic) [6]. Their relatively low sensitivity, the time required for their performance, and patient inconvenience make them unlikely to become point-of-care screening tests.

Oral glucose tolerance tests (OGTTs), are a mainstay for assessing insulin sensitivity [21-25] in the non-invasive diagnosis of impaired glucose tolerance (IGT) and diabetes mellitus. A modified OGTT uses a 75 mg glucose load and measures glucose and insulin at various intervals over two to four hours. OGTT provides information on beta cell secretion and peripheral insulin action. Insulin sensitivity has been assessed by calculating insulin area under the curve (AUC_insulin_), by the AUC_glucose_/AUC_insulin _ratio, and by an insulin sensitivity index (ISI) that uses glucose and insulin values from 0 and 180 min in a mathematical formula. A more qualitative assessment of insulin resistance is the observation of one or more insulin values exceeding an upper, normal, limit at appropriate intervals [25].

The search for simple and inexpensive quantitative tools to evaluate insulin sensitivity has led to development of fasting state (homeostatic) assessments. These tests are based on fasting glucose and fasting insulin, and a calculation to assess insulin sensitivity and beta cell function. The homeostasis model assessment (HOMA) and quantitative insulin sensitivity check index (QUICKI) have been devised, [3,6,22] and may be applied to normoglycemic and hyperglycemic patients. Fasting insulin (I0), and the glucose/insulin (G/I) ratio are inexpensive assays for calculations of insulin resistance [6]. We found a high correlation between the QUICKI score and the PDR at 120 minutes (before exercise), peak time (before exercise), and PDR 90 minutes (post exercise).

Homeostatic model assessment (HOMA) has been widely employed in clinical research to assess insulin sensitivity. Unlike the I0 and G/I ratio, HOMA calculations compensate for fasting hyperglycemia [6]. HOMA values correlate well with the results of clamp techniques, and have been frequently used to assess changes in insulin sensitivity after treatment [26,27]. However, this model assumes that beta cell function is normal and does not apply to patients with type 2 diabetes. One of the weaknesses of these models is that they assume the relationship between glucose and insulin is linear, when in fact it's parabolic. Another weakness is that they assume that beta cell function is normal and do not apply to patients with type 2 diabetes. These indices of insulin resistance necessitate serum insulin and glucose measurements, may require complex calculations, and have not yet made major inroads into general medical practice. None of these can be performed at the point-of-care, and their sensitivity varies over patient populations and BMIs.

Our pre exercise data shows high correlations between HOMA IR 90 min and PDR 90. Our post exercise data shows high correlations between HOMA B 120 and CPDR 30 min, between HOMA IR 60 min and peak time post exercise, and between HOMA B 150 min and PDR 150 min.

Previous studies suggested the use of GBT in patients with diabetes [28]. A comparison of the ^13^C GBT using mass spectrometry with the hyperinsulinemic-euglycemic clamp to determine insulin resistance has been performed, and suggests a high correlation between the results of the two tests [28]. High correlations between ^13^C GBT parameters and glucose metabolism and insulin sensitivity indices from insulin clamp measurements were described. The magnitudes of these correlations compared favourably with QUICKI and were superior to the homeostasis model assessment. However, the use of mass spectrometer in this setting is cumbersome and does not enable the use of the test as a daily decision making tool.

GBT using an office-based device offers several advantages over the currently available techniques for assessment of insulin resistance. It is a non-invasive, point-of-care test. It is not operator dependent, and its sensitivity seems higher than the currently used tests. Currently available methods are either invasive, or not sensitive enough to detect insulin resistance at early stages, or to follow-up treatment. Target populations for screening may include patients with metabolic syndrome, pregnant women, patients with NAFLD, patients at early stages of diabetes, and chronic HCV patients without overt diabetes [29-34].

Although we studied a relative small population of subjects, the data of the present study suggests that the GBT can serve as a non-invasive tool for dynamic evaluation of glucose metabolism, for early diagnosis and for follow-up of patients in groups at high-risk of insulin resistance.

## Abbreviations

IR: Insulin resistance; OGTT: Oral glucose tolerance test; GBT: Glucose breath test; MBT: methacetin breath test; BMI: body mass index; SD: standard deviation; PDR: percent dose recovered; CPDR: cumulative percent dose recovered; ROC: receiver operating characteristic; AUC: area under the curve; HOMA: Homeostasis Model Assessment; QUICKI: quantitative insulin sensitivity check index.

## Competing interests

Yaron Ilan is a Medical Director at Exalenz Biosciences, Israel.

## Authors' contributions

MM: Designed the trail protocol and wrote the manuscript. GL: revised the manuscript. AT: helped to design the trail protocol and analyzed the study results. RI: revised the manuscript. IY: participated in the protocol design, wrote and revised the manuscript. All authors read and approved the final manuscript.
